# Epidemiological models are important tools for guiding COVID-19 interventions

**DOI:** 10.1186/s12916-020-01628-4

**Published:** 2020-05-25

**Authors:** Robin N. Thompson

**Affiliations:** 1 Mathematical Institute, University of Oxford, Woodstock Road, Oxford, OX2 6GG UK; 2 Christ Church, University of Oxford, St Aldates, Oxford, OX1 1DP UK

**Keywords:** COVID-19, Novel coronavirus, SARS-CoV-2, Mathematical modelling, Compartmental models, Forecasting, Non-pharmaceutical interventions, Disease control, Flatten the curve, Lockdown

## Background

The coronavirus disease 2019 (COVID-19) pandemic has been responsible for over three million reported cases worldwide, including more than 200,000 deaths (as of 1 May 2020). In the UK, mathematical models have been employed to inform policy responses, particularly by using model simulations to predict the effects of different non-pharmaceutical interventions (NPIs) [1, 2].

In this article, we discuss epidemiological modelling using a simple model as an example, and highlight an extended model by Davies et al. [1] that has been used to explore the effects of NPIs in the UK. The findings of the extended model were presented to the Scientific Pandemic Influenza Group on Modelling, which is reporting to the Scientific Advisory Group for Emergencies (SAGE). SAGE in turn presents scientific advice to UK government decision makers. We discuss the authors’ results about strategies for intervening to maintain intensive care unit (ICU) bed demand below the number of ICU beds in the UK.

## Epidemiological model

The general framework underlying the study by Davies et al. [1] is compartmental modelling, in which individuals are categorised according to their infection or symptom status [3]. The prototypical compartmental model is the Susceptible-Infectious-Removed (SIR) model,

1 $$ \frac{dS}{dt}=-\beta SI,\kern0.5em \frac{dI}{dt}=\beta SI-\mu I,\kern0.5em \frac{dR}{dt}=\mu I. $$

**Fig. 1:**
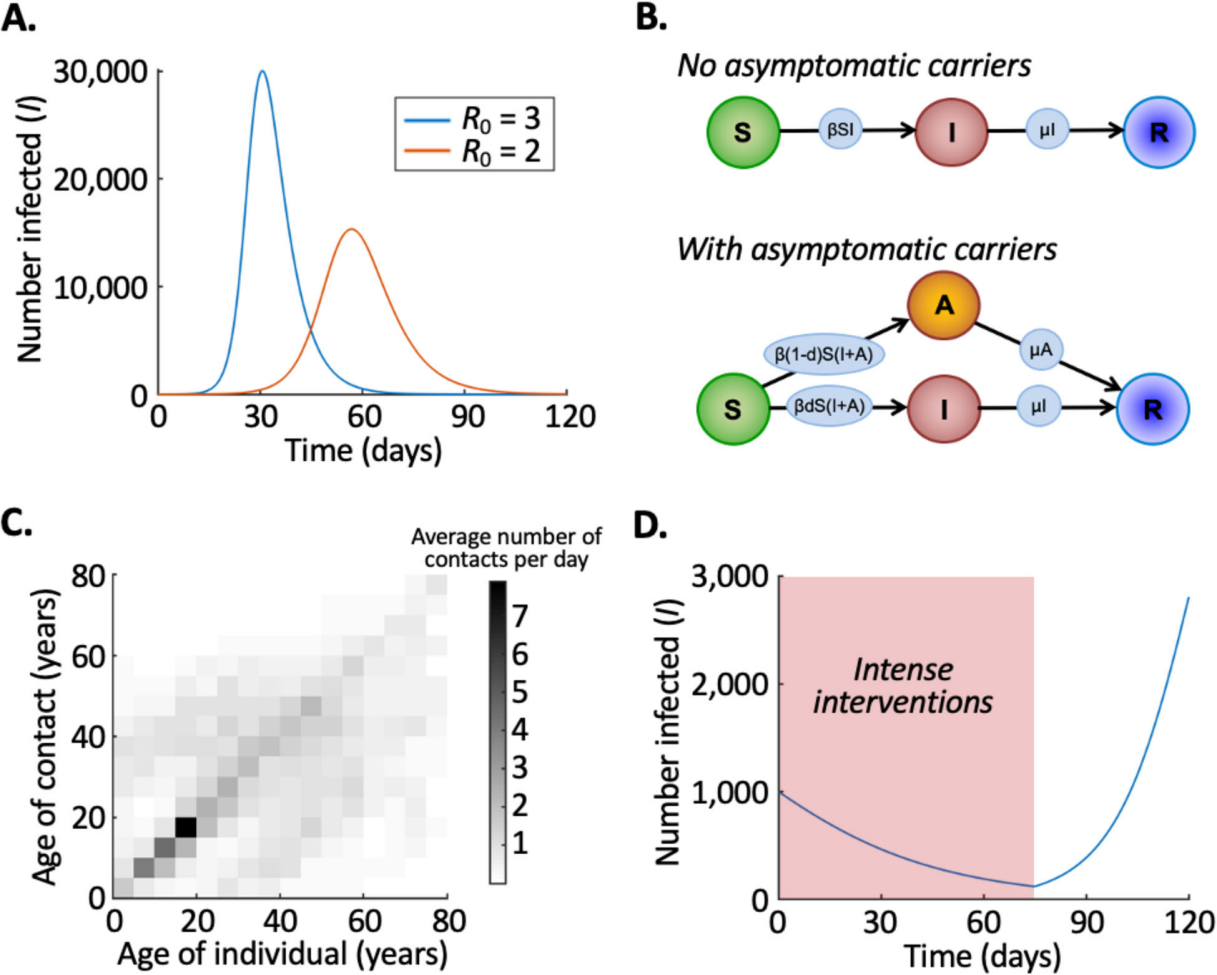
Mathematical models are a useful tool for exploring the potential effects of NPIs against COVID-19. **a** Reducing transmission leads to a “flattening” of the epidemic curve, whereby the peak number of simultaneously infected individuals is smaller and the peak occurs later. **b**, **c** Simple models such as the SIR model can be extended to include features such as asymptomatic infectious individuals (**b**) and different contact rates between individuals of different ages (**c**). **d** When intense interventions are removed, case numbers may begin to increase again. In **a**, the numerical solution of the SIR model (system of Eq.s (1)) is shown for high transmissibility (*R*_0_ = 3, blue line) and low transmissibility (*R*_0_ = 2, red line), starting with *S* = 99,999, *I* = 1 and *R* = 0. In **c**, data show the average numbers of daily contacts that an individual in the age group on the *x*-axis has with contacts in the age group on the *y*-axis, in the UK under normal circumstances [4]. Ages are binned into 5-year intervals (with individuals and contacts who are over 80 years old included in the 75–80 age group). In **d**, the numerical solution of the SIR model (system of Eq.s (1)) is shown with *R*_0_ = 0.9 for all times *t* ≤ 75 days, and *R*_0_ = 1.5 for all times *t* > 75 days, starting with *S* = 99,000, *I* = 1000 and *R* = 0. In **a** and **d**, the infectious period is set to be 1/*μ* = 5 days

The parameter *β* sets the infection rate, and the average infectious period is 1/*μ* days. The basic reproduction number, ${R}_0=\frac{\beta N}{\mu }$, represents the expected number of individuals that a single infectious host will infect if introduced into a population of *N* susceptible hosts. The SIR model can be solved numerically (Fig. 1a).

The compartmental model by Davies et al. [1] includes a number of extensions to the basic SIR model. Their model is stochastic, including randomness that is inherent in real-world outbreaks. Individuals were aggregated into 186 regions across the UK, and transmission was simulated in each region. Individuals with subclinical infections (i.e. who never develop clear symptoms) were assumed to be less infectious than individuals who develop clear symptoms (Fig. 1b indicates, using the SIR model as an example, how “asymptomatic carriers” can be included in compartmental models). Data show that interactions vary between individuals of different ages (Fig. 1c), and so Davies et al. [1] divided the UK population of 66.4 million people into 5-year age groups with different infection rates between groups. The number of individuals requiring hospitalisation and the number of deaths in each age group were assumed to be fractions of the total number of cases, with hospitalisation and death being most likely for elderly cases.

## Effects of control measures

Public health measures in the UK have aimed to “flatten the curve”, namely, (i) reduce the maximum number of individuals simultaneously requiring treatment and (ii) push back the time at which the maximum number of individuals simultaneously requiring treatment occurs. This reduces the maximum number of severe cases requiring treatment at any single time and gives healthcare services longer to increase their capacity to treat (e.g. obtain more ventilators and increase ICU bed availability). The SIR model can be used to demonstrate the principle that a reduction in transmission can delay, and reduce the height of, the epidemic peak (Fig. 1a).

For the extended model by Davies et al. [1], a similar idea applies, but the authors found that short-term implementation of interventions was unlikely to reduce the number of individuals requiring ICU beds below capacity at all times, unless a full “lockdown” was introduced. The authors predicted that, in the absence of interventions, 85% (68–96%) of individuals in the UK would be infected during the epidemic, and peak ICU bed demand would be between 25 and 80 times higher than standard ICU capacity. If a package of mitigation measures (stopping short of a lockdown, but including school closures, social distancing, shielding of high-risk individuals and self-isolation of symptomatic hosts) was implemented in the model for a period of 12 weeks, then the total number of cases decreased substantially compared to the scenario with no interventions and the peak of the epidemic was delayed by 3–8 weeks. However, under these short-term mitigation measures, critical cases were expected to overwhelm healthcare services. Based partly on the results of modelling analyses like that one, a lockdown was introduced in the UK on 23 March 2020, and transmission reduced significantly.

## Sustainable implementation of lockdowns

As of 1 May 2020, the UK epidemic appears to have peaked and attention is now turning to when and how the lockdown can be lifted. Ideally, some interventions can be removed while maintaining transmission at a low level. However, since all aspects of the lockdown were introduced within only a few days, it is challenging to identify the relative effects of each intervention, thereby making it hard to infer whether or not specific measures can be removed safely. Consequently, when the lockdown is relaxed, it is possible that daily case numbers will increase again (Fig. 1d).

Davies et al. [1] demonstrated that, if numbers of incident cases rebound, interventions can be reintroduced to reduce transmission again. When interventions are reintroduced, there is a delay before the reduction in transmission is reflected in case numbers. However, by implementing intermittent lockdowns, the numbers of severely ill patients requiring ICU beds at any single time can be managed. For example, a strategy of implementing lockdown whenever ICU bed usage increases above 1000 beds, and then removing it whenever bed usage falls below that threshold, leads to a predicted peak bed usage of 5000 (3200–39,000).

## Discussion

Mathematical models are being used to guide UK policy during the COVID-19 epidemic. Modelling analyses demonstrated the principle that NPIs could delay the peak of the epidemic and reduce the peak number of cases. However, to ensure that healthcare services were able to cope, substantial interventions were required [1, 2].

Going forwards, the key challenge is to decide when and how to leave lockdown. Ideally, some interventions could be removed with only a small impact on transmission, thereby avoiding a resurgence in cases that overwhelms healthcare services. However, identifying which interventions can be removed safely is challenging and a rebound is possible. Davies et al. [1] showed that, if a rebound occurs, applying a temporary lockdown repeatedly can allow health services to remain effective (similar results were found by [2]). Reintroducing interventions should not be the goal, but may end up being necessary.

To reduce the risk that measures need to be reintroduced, interventions must be removed gradually. Shielding vulnerable subpopulations might be one way to protect high risk individuals. In South Korea, a strategy based on identifying infectious individuals, and tracing and isolating contacts, has allowed the epidemic to be managed with only 249 COVID-19 deaths in that country (as of 1 May 2020). Similar principles could allow the UK epidemic to be contained when the lockdown is relaxed [5], until an effective treatment is found, a vaccine is developed or a sustainable approach for living with the virus is identified.

## Conclusions

Mathematical models are a key tool for guiding public health measures, and outputs from epidemiological modelling analyses should be considered alongside numerous factors (such as potential economic and mental health effects of interventions) when deciding how to intervene. Perfect data are not available, so modelling requires assumptions (e.g. Davies et al. [1] made assumptions about the effects of different interventions on contact rates between hosts). Nonetheless, despite unavoidable uncertainties, models can demonstrate important principles about outbreaks and determine which interventions are most likely to reduce case numbers effectively. Models demonstrated the need for the current lockdown, and modelling must remain a key tool for informing policy as the lockdown in the UK is relaxed.

## Data Availability

Not applicable.

## Abbreviations

COVID-19: Coronavirus disease 2019
ICU: Intensive care unit
NPIs: Non-pharmaceutical interventions
SAGE: Scientific Advisory Group for Emergencies
SIR model: Susceptible-Infectious-Removed model
